# Acute and Chronic Mental Stress both Influence Levels of Neurotransmitter Precursor Amino Acids and Derived Biogenic Amines

**DOI:** 10.3390/brainsci10060322

**Published:** 2020-05-26

**Authors:** Katharina Hüfner, Matyas Galffy, Jonas Egeter, Johannes M. Giesinger, Kathrin Arnhard, Herbert Oberacher, Johanna M. Gostner, Dietmar Fuchs, Barbara Sperner-Unterweger

**Affiliations:** 1 Division of Psychiatry II, Department of Psychiatry, Psychotherapy and Psychosomatics, Medical University Innsbruck, Anichstr. 35, 6020 Innsbruck, Austria; matyas.galffy@i-med.ac.at (M.G.); jonas.egeter@tirol-kliniken.at (J.E.); johannes.giesinger@i-med.ac.at (J.M.G.); barbara.sperner-unterweger@i-med.ac.at (B.S.-U.); 2 Institute of Legal Medicine and Core Facility Metabolomics, Medical University Innsbruck, Muellerstrasse 44, 6020 Innsbruck, Austria; kathrin.arnhard@roche.com (K.A.); herbert.oberacher@i-med.ac.at (H.O.); 3 Institute of Medicinal Biochemistry, Biocenter, Medical University Innsbruck, 6020 Innsbruck, Austria; johanna.gostner@i-med.ac.at; 4 Institute of Biological Chemistry, Biocenter, Medical University Innsbruck, 6020 Innsbruck, Austria; Dietmar.Fuchs@i-med.ac.at

**Keywords:** phenylalanine, tyrosine, kynurenine, tryptophan, neurotransmitter precursor amino acids, mental stress, tetrahydrobiopterin, biogenic amines, nitrite, neopterin, stress hormones

## Abstract

Acute and chronic mental stress are both linked to somatic and psychiatric morbidity, however, the neurobiological pathways of these associations are still not fully elucidated. Mental stress is known to be immunomodulatory, which is one of the basic concepts of psychoneuroimmunology. In the present study, neurotransmitter precursor amino acid levels and derived biogenic amines were analyzed prior to and at 0, 30 and 60 min following an acute mental stress test (with/without chronic mental stress) in 53 healthy subjects. Psychometric measurements of mental stress, depression and anxiety were collected. Kynurenine/tryptophan was influenced by the factor acute mental stress (KYN/TRP increase), no influence of the factor chronic mental stress or any interaction was found. Phenylalanine/tyrosine was influenced by the factor acute mental stress (PHE/TYR increase) as well as by chronic mental stress (PHE/TYR decrease). Interactions were not significant. KYN/TRP correlated with state anxiety values, while PHE/TYR correlated negatively with chronic stress parameters. Kynurenic acid was significantly reduced in the acute and quinolinic acid in the chronic mental stress condition. In conclusion, neurotransmitter precursor amino acid levels and derived biogenic amines are influenced by acute and chronic mental stress. Mechanisms beyond direct immunological responses may be relevant for the modulation of neurotransmitter metabolism such as effects on enzyme function through cofactor availability or stress hormones.

## 1. Introduction

“Mental stress is not a vague concept somehow related to the decline in the influence of traditional codes of behaviour, dissatisfaction with the world, or the rising cost of living, but (…) clearly a definable biological and medical phenomenon the mechanisms of which can be objectively identified” [1]. The term “stress” has often been used to describe a black box hiding the mechanisms promoting mental health problems [2,3] but actually there are some exact biological mechanisms underlying mental stress and its association with mental and physical disease. However, not all mechanisms have been fully understood and investigated to date. For example, the association between mental stress and ischemic heart disease is undisputed by most, but probably a complicated and complex one [4,5].

The hypothalamic-pituitary adrenocortical axis (e.g., cortisol) and the sympathetic adrenomedullary system (e.g., adrenaline) are generally considered to be the two key players in the physiological stress response [6,7]. This stress response can affect the immune system e.g., by altering the numbers of circulating natural killer cells or levels of cytokines [8,9]. The availability of neurotransmitter precursor amino acids, which are associated with the levels of neurotransmitters in the brain, can be influenced either via stress-induced immune changes or by the direct action of stress hormones on synthesizing enzymes.

**Figure 1:**
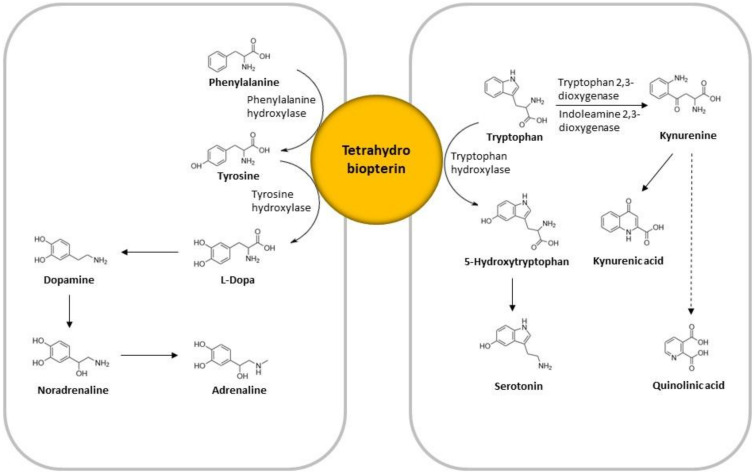
Graphical depiction of the amino acid neurotransmitter pathways analyzed in the current study. The central role of tetrahydrobiopterin (BH4) in both pathways is emphasized.

Tryptophan metabolism is influenced by pro-inflammatory cytokines, which enhance the activity of indoleamine 2,3-dioxygenase (IDO-1) or by stress hormones, which can activate tryptophan (TRP) 2,3-dioxygenase (TDO) [10,11,12]. Both enzymes are involved in the breakdown of TRP to kynurenine (KYN) [13]. In addition, IDO-2 can catalyze this reaction but has a much lower affinity to TRP than IDO-1 and its specific contributions to physiology are largely unknown [14]. The resulting TRP depletion leads to lower levels of serotonin (5-HT), which could, in turn, increase the risk for depressive symptoms and anxiety [15] (Figure 1). Additionally, breakdown of KYN to kynurenic (KA) and quinolinic (QA) acid generates two neuroactive substances [16]: KA can reduce glutamate release as well as the release of dopamine, both of which can contribute to cognitive dysfunction. By contrast, QA, through activation of the N-Methyl-D-aspartate receptor, can increase glutamate release as well as lead to lipid peroxidation, thus contributing to excitotoxicity, oxidative stress, and ultimately neurodegeneration [17]. KYN/TRP is considered a marker of IDO and TDO activity (Figure 1) [18].

The availability of the enzyme co-factor tetrahydrobiopterin (BH4) is essential for PHE catabolism involving PHE hydroxylase (PAH) [19], and also for serotonin synthesis. TRP hydroxylases (TPH), which convert TRP to 5-hydroxytryptophan (5-HTP), the precursor of 5-HT, are dependent on BH4 [17]. BH4 availability can be influenced by inflammatory stimuli such as interferon gamma, which increases BH4 synthesis [20]. While most cell types preferentially produce BH4, this biosynthetic pathway is directed towards neopterin formation in human macrophages and dendritic cells, due to the deficiency of a downstream processing enzyme. Reactive oxygen species (ROS) reduce the availability of BH4 [21], thereby influencing catecholamine and serotonin synthesis. A deficiency in BH4 can cause severe neurological issues related to a toxic accumulation of PHE and lead to systemic deficiencies of dopamine, norepinephrine, and epinephrine. TYR hydroxylase (TH), which converts TYR into L-3,4-dihydroxyphenylalanine (L-Dopa), also depends on BH4. PHE/TYR ratio is utilized as an estimate of PAH activity (Figure 1).

Up to now, it has been barely investigated whether mental stress influences the availability of neurotransmitter precursor amino acids and, if so, which mechanisms might be important in this association. Therefore, we evaluated PHE/TYR, KYN/TRP, KA and QA levels in serum samples of a very homogenous population of somatically and mentally healthy individuals prior to and following an acute mental stress test once in the presence and once in the absence of chronic mental stress. We investigated whether the changes induced by acute and chronic mental stress would induce differential or similar changes in levels of neurotransmitter precursor amino acids and derived biogenic amines and if chronic mental stress would lead to a sensitisation towards acute mental stress.

## 2. Methods

### 2.1. Ethics Statement

The study was approved by the ethics committee of the Medical University Innsbruck, Austria. Informed written consent was obtained from all participants prior to inclusion in the study.

### 2.2. Study Design

The presented data are part of a larger study evaluating the effect of mental stress on biological markers, namely immunological parameters (Koudovouh-Tripp unpublished data). We assessed the 53 study participants at two time points: with and without the presence of chronic mental stress. The order of the study conditions was variable. The chronic mental stress condition consisted of a major university exam with an average three-month preparation time. At each study visit, a standardized acute mental stress test was carried out. Blood was collected at four time points: at baseline after 30 min of rest after arrival to the study location (T0), immediately after the acute mental stress test (T1), 30 min post-acute mental stress (T2) and 60 min post-acute mental stress (T3).

### 2.3. Participants

Power analysis was done for a repeated measures analysis of variance with eight repeated measurements. A sample size of *N* = 50 allows to detect a difference of 0.44 standard deviations between the conditions with/without chronic mental stress (with four measurements each) and a difference of 0.34 standard deviations between the conditions acute mental stress (four time points with two measurements each) with alpha = 0.05 and beta = 0.20 (correlations between repeated measures of 0.50). All participants were mentally and physically healthy medical students who were recruited through our university program. Inclusion criteria: male medical students, no mental disorder, no somatic disorder requiring medical attention (specifically no active inflammatory disorder), non-smoker, no adherence to any specific diet, no caffeinated or alcoholic beverages prior to the assessment, no excessive exercise or sleep deprivation 24 h before the assessment, no immunomodulatory medication in the 14 days prior to the assessment. The participants were excluded if a psychiatric or somatic condition requiring medical attention was present. Mental health was screened for using the German Version of the Mini International Neuropsychiatric Interview 5.0 M.I.N.I. [22]. Somatic health was assessed by questions about previous health conditions. Thyroid replacement therapy was the only medication allowed at the time of the study. The following general, participant-related parameters were collected: sociodemographic data, physical activity (self-administrable German version of the International Physical Activity Questionnaire (IPAQ) [23]) and sleep quality (German version of the Pittsburgh Sleep Quality Index (PSQI) [24]). Enrollment took place between March 2013 and March 2016.

### 2.4. Acute Stress Test

The acute stress test consisted of 2 × 2.5 min of the ‘Stroop’ colour and word test followed by 2 × 2.5 min of a standardized arithmetic test [25,26]. The pressure on the participants was increased by (1) the examiner wearing a white coat and performing minimal interaction with the participants outside the study protocol, (2) a computer-generated metronome sound, (3) the introduction of social pressure via computer screen instruction reading, for example, “it is very important that you answer as many of the trials correctly as possible”, or “your score will be recorded and compared to the score of other individuals performing this test”. In the ‘Stroop’ task, an additional stress factor was that participants could not perform the test at their own pace, but must adapt to the computer-generated pace, and in the mental arithmetic task, stress was increased by the examiner pointing out wrong answers and asking to restart the calculation series after each wrong answer.

### 2.5. Assessment of Stress-Related Psychometric Parameters

Screening for depressive symptoms was done with the “Becks’s Depression Inventory” (BDI) [27]. The “Perceived Stress Scale 14” (PSS-14) [28], and the “Trier Inventory of Chronic Stress” (TICS) [29] were used for stress-related assessments. All are self-reporting instruments and digitalized versions were used which were completed by the study participants via an online link in the days prior to each study visit. The “State-Trait Anxiety Inventory” (STAI) was used to assess anxiety symptoms on the day of the study via a paper and pencil version [30]. Participants rated their subjective amount of mental stress from each acute stress task on a five-point Likert Scale following the acute mental stress test.

### 2.6. Blood Sampling

A peripheral venous catheter was inserted into the antecubital vein of the non-dominant hand in all participants. Participants rested for 30 min following the insertion before the first (resting, T0) blood sample was drawn. The samples were collected in commercially available tubes (Sarstedt, Vienna, Austria). Routine blood parameters were determined at the hospital central laboratory including TSH and full blood l count. Aliquots of serum were processed immediately and shock frozen in liquid nitrogen and stored at −80 °C until use. The first blood draw (T0) was done between 8:00 and 8:30 a.m. in all participants.

### 2.7. Neurotransmitter Precursor Amino Acid Analysis

Neopterin concentrations were measured by enzyme-linked immunosorbent assay (BRAHMS Diagnostics, Berlin, Germany). TRP and KYN serum concentrations, as well as concentrations of PHE and TYR, were determined by high-performance liquid chromatography, as described elsewhere [21,31]. The ratios of KYN/TRP and PHE/TYR were calculated as indices of IDO and PHA activity, respectively [32]. Serum nitrite concentrations, which are considered surrogate markers for nitric oxide, were measured via the Griess reaction assay [33]. KA and QA were determined by mass spectrometry as described previously; for technical reasons only samples from a randomly selected subset of 31 participants were analyzed [34].

### 2.8. Statistical Analysis

Linear mixed model analysis was performed for testing differences in measured serum parameters across conditions, i.e., the main effects of acute mental stress (T0, T1, T2, T3) and chronic mental stress (Yes vs No). The model investigated the main effects of acute and chronic mental stress as well as their interaction effect on neopterin, nitrite and neurotransmitter precursor amino acid levels as well as derived biogenic amines. To account for correlation between repeated assessments, we used a 1st order autoregressive covariance matrix and included an additional random intercept on participant level. Variables with non-normal distribution were log-transformed to approximate their distribution of normality. In case of a significant impact of sleep (PSQI), the order of examinations (chronic mental stress or no chronic mental stress condition first) or physical activity (IPAQ, metabolic units MET), we adjusted for these variables. Non-significant results were removed in a stepwise procedure. Sample characteristics are given as means and standard deviations. The results of lifestyle and stress-related psychometric assessments and neopterin, nitrite, neurotransmitter precursor amino acids as well as derived biogenic amines, were expressed as means and confidence intervals. Non-parametric analyses were performed to test for differences in lifestyle and stress-related psychometric parameters between the two study visits. Correlation analyses tested for a correlation of neurotransmitter precursor amino acid levels with STAI state, BDI depression values and TICS chronic stress values using Spearman rank-order correlation analyses. *p* < 0.05 was considered significant in these analyses. All analyses were done in SPSS 20.0.

## 3. Results

### 3.1. Study Population

A very homogenous sample of 53 mentally and physically healthy male medical students participated in the study. The mean age was 23.0 years ± 2.4 (mean ± SD), the mean BMI was 22.7 + 2.3 kg/m^2^ (mean ± SD). Participants had to adhere to strict behavioural criteria prior to the study visit such as avoiding caffeine, alcohol, exercise and sleep deprivation in the 24 h prior to the study. A special diet, smoking or medication (thyroid replacement therapy exempt) were not allowed. The mean time to the exam was 11.8 ± 6.1 days (mean ± SD) in the chronic mental stress condition and 100.1 ± 51.0 days (mean ± SD) in the condition without chronic mental stress.

### 3.2. Psychometric Stress Assessment

Perceived stress was higher in the chronic mental stress condition compared to no chronic mental stress for the Perceived Stress Scale (PSS; Wilcoxon test, *p* < 0.001) as well as the Trier Inventory of Chronic Stress (TICS; Wilcoxon test, *p* = 0.004). State anxiety (STAI state, Wilcoxon test, *p* = 0.001) and depressive symptoms (BDI, Wilcoxon test, *p* = 0.02) were also higher in the chronic mental stress condition, however, values for both conditions were still quite low (Table 1).

### 3.3. The Effect of Mental Stress on Serum Neopterin and Nitrite Levels

The factor “acute mental stress” (linear mixed model, *p* = 0.243) did not influence neopterin levels, while “chronic mental stress” (linear mixed model, *p* = 0.015) was associated with higher neopterin levels (Table 2 and Table 3). The interaction of “acute mental stress” and “chronic mental stress” on neopterin levels was not significant. Over all analyzed timepoints, neopterin levels correlated with PHE/TYR (Spearman rank-order correlations, *r* = 0.195; *p* < 0.001) and KYN/TRP (Spearman rank-order correlations, *r* = 0.299; *p* < 0.001). Nitrite levels were not influenced by the factor “acute mental stress” (linear mixed model, *p* = 0.269) but the factor “chronic mental stress” lead to decreased nitrite levels (linear mixed model, *p* = 0.017), while the interaction of acute and chronic mental stress was not significant.

### 3.4. The Effect of Acute Mental Stress on Neurotransmitter Precursor Amino Acids

**Figure 2:**
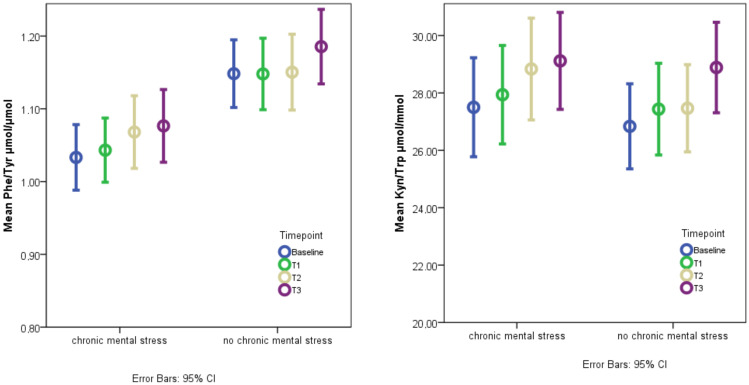
Plots of observed values of neurotransmitter precursor amino acid levels. Plots represent means and 95% confidence intervals (CI) of the observed values. *n* = 53. Adjusted *p*-values from the linear mixed model can be found in Table 2 and Table 3. Baseline = T0 after 30 min of rest, T1 = immediately after acute mental stress test, T2 = 30 min after acute mental stress test, T3 = 60 min after acute mental stress test.

The factor “acute mental stress” lead to a significant increase in PHE/TYR (linear mixed model, *p* = 0.023), which was mainly due to TYR decreases from T0 to T3. PHE also decreased in response to acute stress but to a lesser amount (Figure 2, Table 2, Supplemental Table S1). KYN/TRP was significantly increased by the factor “acute mental stress (linear mixed model, *p* = 0.003)”, which was mainly due to TRP decreases. KYN was not significantly altered by the factor “acute mental stress” (Figure 2, Table 2, Supplemental Table S1).

### 3.5. The Effect of Chronic Mental Stress on Neurotransmitter Precursor Amino Acids

PHE/TYR was significantly decreased in the chronic mental stress condition (linear mixed model, *p* < 0.001) compared to the condition without chronic mental stress. This was due to decreases in PHE concentrations; there was no significant effect on TYR (Table 3, Supplemental Table S2). KYN/TRP was not significantly influenced by the factor “chronic mental stress” (linear mixed model, *p* = 0.171) since both KYN and TRP were increased by chronic mental stress (Table 3, Supplemental Table S2). Interactions between “acute mental stress” and “chronic mental stress” on PHE/TYR and KYN/TRP were not significant.

### 3.6. The Effect of Acute and Chronic Mental Stress on QA and KA Levels

KA and QA were analyzed in a subset of 31 randomly selected study participants. KA was reduced (linear mixed model, *p* = 0.002) but QA was not significantly altered by the factor “acute mental stress” while the factor “chronic mental stress” lead to significantly reduced QA levels (linear mixed model, *p* = 0.002), while KA was left unaffected. The interaction between acute and chronic mental stress was not significant either for KA or QA (Table 2 and Table 3).

### 3.7. Correlation Analyses

KYN/TRP levels correlated positively with state anxiety (STAI state, Spearman rank order correlations, *r* = 0.116, *p* = 0.018), PHE/TYR correlated negatively with psychometric measurements of chronic stress (PSS, Spearman rank-order correlations, *r* = −0.099, *p* = 0.044; Trier Inventory of Chronic Stress, *r* = 0.112, *p* = 0.021).

## 4. Discussion

In this study, we evaluated the effect of acute and chronic mental stress on neurotransmitter precursor amino acid levels and derived biogenic amines in a very homogenous population of healthy students. We observed increases in PHE/TYR and KYN/TRP due to acute mental stress and decreases in PHE/TYR due to chronic mental stress, while KA was decreased by acute mental stress and QA by chronic mental stress. The allostatic load in this population was not excessive: while we observed small but significant differences in the psychometric chronic stress assessments, values for mental stress, depression and anxiety were still very low, indicating good coping strategies and no allosteric overload. We discuss the results against the hypothesis, that short term mental stress is a survival mechanism modulating the immune response in a beneficial way, while long-term mental stress can suppress or dysregulate the immune balance [35].

### 4.1. PHE/TYR and KYN/TRP and Acute Mental Stress

Acute mental stress might exert its effect through the activation of the adrenaline/noradrenaline system and its effect on enzymes and metabolism. In the present study, acute mental stress lead to an increase in PHE/TYR and KYN/TRP from baseline to 60 min post mental stress. It is unclear how this increase within the 60-minute time window is achieved. It could be due to a deployment of storages and increased release of amino acids and catabolites in the bloodstream. Resting enzymes could be activated to increase production of TYR and KYN. The *de novo* synthesis of enzymes involved in neurotransmitter amino acids metabolism seems questionable within this time frame.

Stressful experiences often result in wounding or infection (if looked at from a basic evolutionary perspective of the “fight and flight” response to acute stress), hence “immunoenhancement, rather than immunosuppression, would be adaptive during short-term stress because it is unlikely that millions of years of evolution would select for a system exquisitely sculpted to escape the jaws and claws of a lion only to succumb to wounds and microbes“ [36]. This immune stimulating effect could explain the observed higher levels in PHE/TYR and KYN/TRP, which are commonly regarded as a sign of immune activation.

### 4.2. The Effect of Chronic Mental Stress on Neurotransmitter Precursor Amino Acids

PHE/TYR was reduced due to chronic mental stress, mainly because of lower PHE concentrations. From an immunological point of view, on the one hand this may suggest that there was no chronic low-grade inflammation in the chronic mental stress condition as has been described e.g., for individuals with cancer or depression [37], but rather this could be a sign of immunological suppression as has been described previously in such young and otherwise healthy samples [36]. On the other hand, in this healthy and homogenous collective of participants, it is also possible that the observed PHE/TYR decrease is related to a direct biochemical mechanism (increased neopterin and BH4). Nitrite as a surrogate marker for nitric oxide, was also reduced in the chronic mental stress condition paralleling PHE/TYR levels, as a sign of BH4 metabolism [38], while neopterin was increased. This finding underlines a possible involvement of BH4 metabolism in the observed PHE/TYR changes induced by chronic mental stress. BH4 is also important for nitric oxide synthesis and thus involved in oxidative stress; an interaction between nitric oxide and the HPA- axis is increasingly recognized [39]. The concentrations of PHE and TYR were close to the normal range [40]. In populations with “more hits” such as elderly caregivers with cardiovascular disease, this might show very different effects [41]. In the collective evaluated here, the allostatic load was apparently not very high so that there were stable allostatic conditions. Although it might be expected that beneficial effects would be derived from BH4 supplementation in depressive patients, contradictory results have been reported so far [42,43]. BH4 treatment has been reported to be ineffective or, indeed, to be even associated with an elevation of clinical symptoms of depression [44].

KYN/TRP was not affected by chronic mental stress, which fits the hypothesis that no immunological activation or reaction along the KYN axis took place in this cohort. Although glucocorticoids have been shown to induce TDO by several-fold, only moderately decreased tryptophan concentration in liver and brain and moderately elevated plasma kynurenine concentration were observed [45].

### 4.3. QA and KA in Acute and Chronic Mental Stress

KA was reduced due to acute mental stress. It acts as an endogenous competitive antagonist of N-methyl-D-aspartate (NMDA) receptor [46] and thus reduced KA levels can be advantageous for cognitive performance. QA was reduced in chronic mental stress. This might be due to the fact that we examined a healthy population with no deficits in QA degradation and metabolism. Therefore, no toxic metabolites were able to accumulate and no signs of neurodegeneration or dysfunction occurred. QA is often described as neurotoxic primarily due to activation of the NMDA receptor and free radical production [47,48]. We found anecdotally that the students were quite well prepared for the exam and not overly anxious when we examined them only a few days prior. No influence of chronic mental stress on KA was observed.

### 4.4. Limitations

In this study, we evaluated the effect of acute mental stress only with a limited follow-up time of 60 min, which did not allow us to observe the recovery of amino acid concentrations following the acute mental stress test. Additionally, since only male subjects were included to avoid influence of hormonal cycle on the parameters, we cannot infer to females from these data, who could potentially be more susceptible to chronic mental stress. Since a mental stress test to elicit acute mental stress was performed in all participants, we cannot separate in this design effects of acute mental stress due to the study situation at the hospital from acute mental stress due to the administered mental stress task.

## 5. Conclusions

Usually, immunology is a strong driving force for PHE/TYR and KYN/TRP metabolism in most circumstances. But in the very immunologically healthy cohort with no signs of low-grade inflammation also, more mechanistic interpretations or direct actions of stress hormones might be of greater relevance. Of course, also a mixture of effects with different interactions and interplays is possible. This could explain some of the conflicting results observed in studies on KYN/TRP and PHE/TYR in neuropsychiatric conditions. It is possible that direct effects of stress hormones, BH4 availability and immunological forces influence KYN/TRP and PHE/TYR in opposite directions. Whether these results are transferrable to patients with mental health disorders will have to be examined in a follow-up study.

## Figures and Tables

**Table 1:** Overview of stress related parameters

Stress Parameter	Chronic Mental Stress	No Chronic Mental Stress	*p*-Value
PSS (range 0–56 points)	18.4 (16.3–20.6)	15.1 (13.4–16.8)	<0.001 *
BDI (range 0–63 points)	3.2 (2.2–4.3)	2.2 (1.5–2.9)	0.020 *
Trait anxiety scale (STAI, range 20–80 points)	47.1 (46.4–47.9)	46.9 (46.2–47.7)	0.811
State anxiety scale (STAI, range 20–80 points)	30.7 (31.2–36.1)	30.2 (28.3–32.0)	<0.001 *
TICS chronic stress (range 0–48 points)	12.7 (10.4–14.9)	10.3 (8.6–12)	0.004 *
Stress from “Stroop” test (5 Point Likert scale)	3.3 (3.1–3.6)	3.1 (2.8–3.3)	0.042 *
Stress from arithmetics test (5 Point Likert scale)	2.5 (2.3–2.8)	2.6 (2.3–2.9)	0.431
Days to exam (days)	11.8 (11–12.7)	100.2 (93.3–107.1)	<0.001 *

Results are given as means (95% confidence interval), Wilcoxon test was used to compare the chronic mental stress condition and the condition without chronic mental stress. Significant *p*-values are indicated by an asterisk. Ranges of the respective tests or subtests are given to aid with interpretation. Abbreviations: PSS = Perceived Stress Scale, STAI = State-Trait Anxiety Scale, BDI = Beck´s Depression Inventory, TICS = Trier Inventory of Chronic Stress, chronic stress subscale.

**Table 2:** The effect of acute mental stress on neurotransmitter precursor amino acid levels, derived biogenic amines, neopterin and nitrite.

	T0	T1	T2	T3	*p*-Value
PHE/TYR (µmol/µmol)	1.09 (1.05–1.13)	1.10 (1.05–1.14)	1.11 (1.07–1.15)	1.13 (1.09–1.17)	*p* = 0.023 *
KYN/TRP (µmol/mmol)	27.1 (25.7–28.6)	27.7 (26.3–29.1)	28.1 (26.7–29.5)	28.9 (27.5–30.3)	*p* = 0.003 *^,2,3^
QA (ng/ml)	58.1 (49.4–66.8)	56.8 (48.1–66.8)	54.6 (45.9–63.3)	54.9 (46.2–63.5)	*p* = 0.530 ^2^
KA (ng/ml)	21.1 (19.4–22.8)	20.4 (18.7–22)	19.5 (17.8–21.2)	18.6 (16.9–20.3)	*p* = 0.002 *
Neopterin (nmol/L)	5.33 (5.01–5.65)	5.29 (4.97–5.61)	5.41 (5.09–5.73)	5.29 (4.97–5.60)	*p* = 0.243 ^1,3^
Nitrite (µmol/L)	23.8 (19.2–28.4)	24.8 (20.2–29.3)	24.3 (19.7–28.9)	24.3 (19.8–28.3)	*p* = 0.269

Results are given as means (95% confidence interval) for *n* = 53 participants (subset of *n* = 31 for KA and QA). A linear mixed model including the factors “acute mental stress” and “chronic mental stress” with co-variables was used for analysis. Statistically significant *p*-values are indicated by an asterisk. The interaction of acute and chronic mental stress was not significant in any of the analyses. ^1^ adjusted for Pittsburgh Sleep Quality Index (PSQI), ^2^ adjusted for International Physical Activity Questionnaire (IPAQ), ^3^ adjusted for order of examinations (chronic mental stress or no mental stress condition first). T0 = baseline. T1 = immediately following acute mental stress test. T2 = 30 min following acute mental stress test. T3 = 60 min following acute mental stress test.

**Table 3:** The effect of chronic mental stress on neurotransmitter precursor amino acid levels, derived biogenic amines, neopterin and nitrite.

	Chronic Mental Stress	No Chronic Mental Stress	*p*-Value
PHE/TYR (µmol/µmol)	1.06 (1.01–1.10)	1.16 (1.12–1.20)	*p* < 0.001 *
KYN/TRP (µmol/mmol)	28.5 (27.0–29.9)	27.5 (26.0–29.0)	*p* = 0.171 ^2^
QA (ng/ml)	48.5 (39.3–57.7)	63.6 (54.5–72.8)	*p* = 0.002 *^,2^
KA (ng/ml)	20.5 (18.7–22.3)	19.3 (17.5–21.1)	*p* = 0.148
Neopterin (nmol/L)	5.50 (5.16–5.83)	5.16 (4.83–5.50)	*p* = 0.015 *^,1,3^
Nitrite (µmol/L)	23.0 (18.2–27.8)	25.6 (20.8–30.4)	*p* = 0.017 *

Results are given as means (95% confidence interval) for *n* = 53 participants (subset of *n* = 31 for KA and QA). A linear mixed model including the factors “acute mental stress” and “chronic mental stress” with co-variables was used for analysis. Statistically significant p-values are indicated by an asterisk. The interaction of acute and chronic mental stress was not significant in any of the analyses. ^1^ adjusted for Pittsburgh Sleep Quality Index (PSQI), ^2^ adjusted for International Physical Activity Questionnaire (IPAQ), ^3^ adjusted for order of examinations (chronic mental stress or no mental stress condition first).

## Supplementary Materials

The following are available online at https://www.mdpi.com/2076-3425/10/6/322/s1, Table S1: The effect of acute mental stress on neurotransmitter precursor amino acid levels, Table S2: The effect of chronic mental stress on neurotransmitter precursor amino acid levels.
