# Supplementary material for: Acute and Chronic Mental Stress both Influence Levels of Neurotransmitter Precursor Amino Acids and Derived Biogenic Amines
Source: Brain Sci. 2020 May 26;10(6):322. doi: 10.3390/brainsci10060322 (PMC7349276; doi:10.3390/brainsci10060322)
Supplement: Supplementary file 1 [file brainsci-10-00322-s001.pdf]

Supplemental materials

Table 1. The effect of acute mental stress on neurotransmitter precursor amino acid levels.

	T0	T1	T2	T3	p-value
PHE (µmol/l)	79.8 (77.2 – 82.3)	76.0 (73.5 – 78.5)	72.6 (70 – 75.1)	68.6 (66.1 – 71.1)	$p < 0.001$*
TYR (µmol/l)	75.3 (70.9 – 79.6)	71.1 (67.0– 75.8)	67.3 (63.1 – 71.9)	62.4 (58.0– 66.7)	$p < 0.001$*
KYN (µmol/l)	2.09 (1.97 – 2.20)	2.06 (1.95 – 2.17)	2.03 (1.92 – 2.14)	2.02 (1.91 – 2.13)	$p = 0.181$[2]
TRP (µmol/l)	76.0 (74.2 – 77.9)	73.8 (71.9 – 75.6)	71.2 (69.3 – 73.0)	68.9 (67.0 – 70.7)	$p < 0.00$ *

Results are given as mean (95% confidence interval) for n=53 participants. A linear mixed model including the factors “acute mental stress” and “chronic mental stress” with co-variables was used for analysis. Statistically significant p-values are indicated by an asterisk. The interaction of acute and chronic mental stress was not significant in any of the analyses. [2] adjusted for International Physical Activity Questionnaire (IPAQ). T0 = baseline. T1 = immediately following acute mental stress test. T2 = 30 minutes following acute mental stress test. T3 = 60 minutes following acute mental stress test.

Table S2. The effect of chronic mental stress on neurotransmitter precursor amino acid levels.

	Chronic mental stress	No chronic mental stress	p-value
PHE (µmol/l)	70.5 (67.8 – 73.2)	77.4 (74.7 – 80.1)	$p < 0.001$*
TYR (µmol/l)	69.2 (64.45– 73.6)	69 (64.5 – 73.6)	$p = 0.923$
KYN (µmol/l)	2.10 (1.98 – 2.22)	2.00 (1.88 – 2.11)	$p = 0.024$*[2]
TRP (µmol/l)	74.6 (72.7 – 76.5)	70.6 (68.4 – 72.3)	$p < 0.001$*

Results are given as mean (95% confidence interval) for n=53 participants. A linear mixed model including the factors “acute mental stress” and “chronic mental stress” with co-variables was used for analysis. Statistically significant p-values are indicated by an asterisk. The interaction of acute and chronic mental stress was not significant in any of the analyses. [2] adjusted for International Physical Activity Questionnaire (IPAQ).
